# Exploring the silent storm

**DOI:** 10.5339/qmj.2024.qitc.3

**Published:** 2024-03-25

**Authors:** Mohamed Elgara, Theeb Osama Sulaiman, Mousa Hussein, Mona Al Langawi

**Affiliations:** 1 Hamad General Hospital, Hamad Medical Corporation, Doha, Qatar Email: melgara@hamad.qa; 2 College of Medicine, Qatar University, Doha, Qatar

**Keywords:** Pulmonary alveolar microlithiasis, SLC34A2, Microliths, Parenchymal lung disease, Sandstorm

## Introduction

Pulmonary alveolar microlithiasis (PAM) is a rare inherited disorder caused by mutations in the recessive gene SLC34A2, which is found on an autosomal chromosome. This gene encodes a phosphate transporter in alveolar cells, resulting in the accumulation of calcium phosphate-rich granules called microliths in the alveoli.^1-3^

## Case Summary

**Figure 1.**
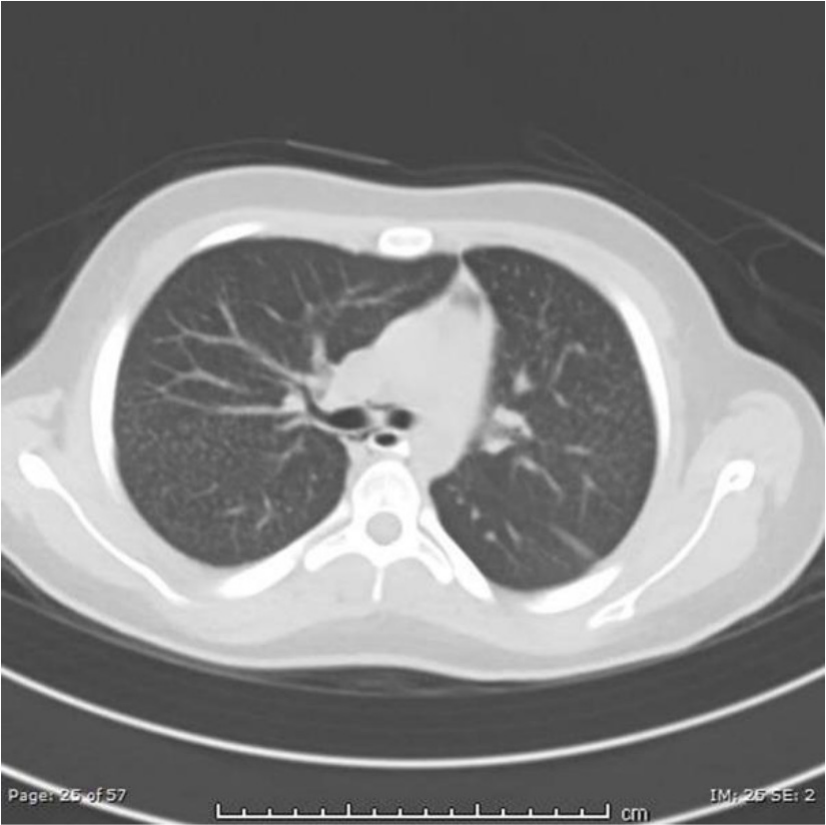
The image shows diffuse nodular densities in both lungs with a centrilobular bronchial distribution and a tree-in-bud appearance.

We present the first four cases of PAM reported in Qatar. The first case involves a 16-year-old male who presented to the emergency department (ED) with sudden chest pain. A chest X-ray showed pneumomediastinum, which required further investigation. A chest CT scan revealed diffuse nodular densities in both lungs with a centrilobular bronchial distribution and a tree-in-bud appearance (Figure 1). He had no previous respiratory symptoms. Subsequently, bronchoscopy with transbronchial biopsy confirmed extensive alveolar laminated microcalcifications and established the diagnosis of PAM.

**Figure 2.**
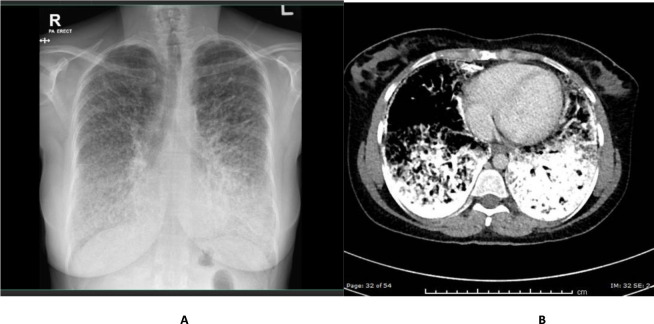
(A) A chest X-ray shows a “sandstorm” appearance of diffuse scattered micronodules with a predilection for the lung bases. (B) Progressive opacification and intense calcification with thickening of the interstitium.

The second case is a 38-year-old Yemeni woman who was presented to the ED with sore throat and fever and diagnosed with follicular tonsillitis. Her chest X-ray is shown in Figure 2A. She denied respiratory symptoms and had no family history of lung disease, occupational exposures, or smoking history. A chest CT (Figure 2B) showed intensive opacification and calcification with interstitial thickening. A bronchoscopy with transbronchial biopsy was recommended to confirm the diagnosis, but she declined.

**Figure 3.**
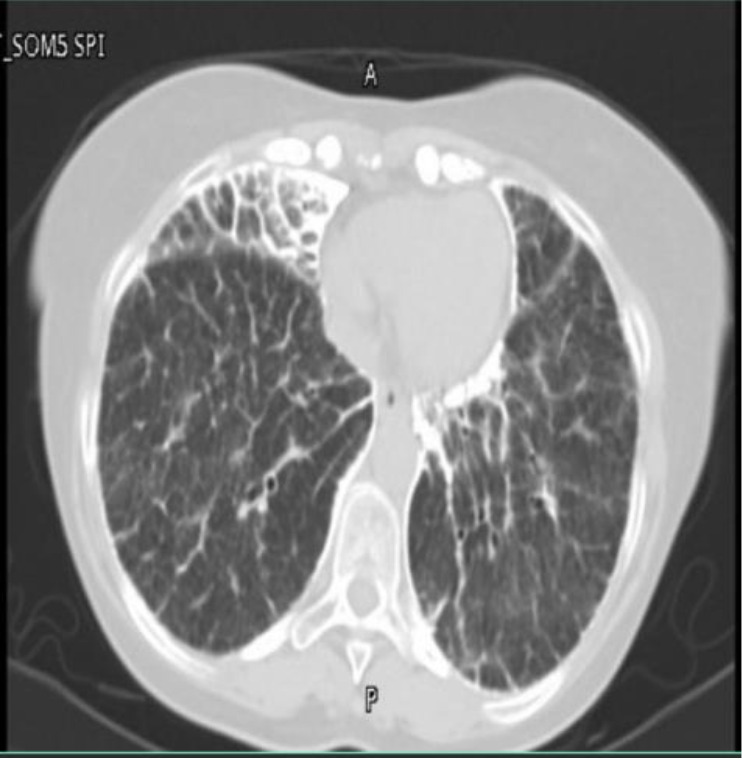
Diffuse interstitial thickening with calcifications and basal predominance.

**Figure 4.**
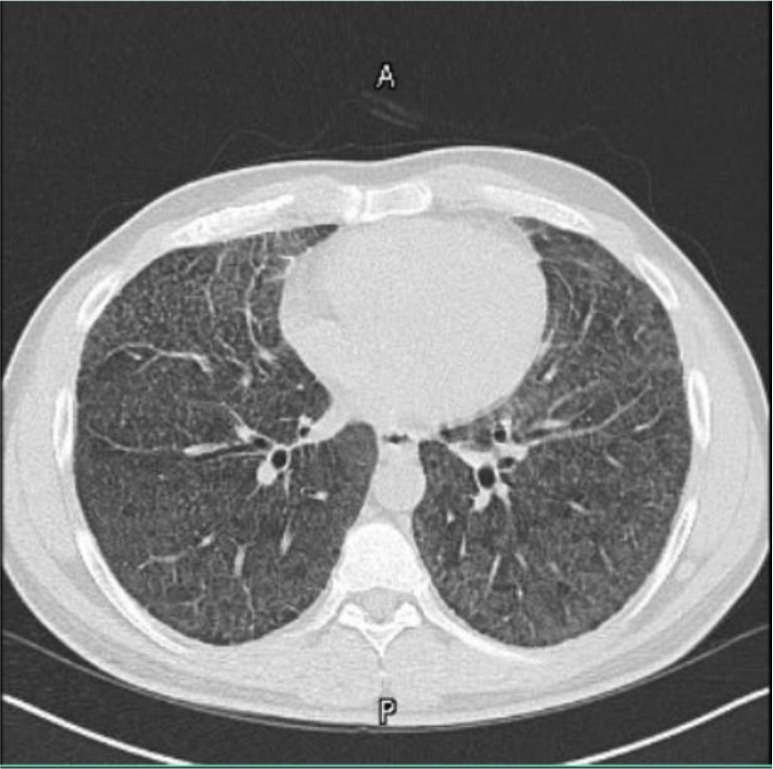
Diffuse calcified micronodular infiltrates with interstitial thickening.

The third and fourth cases involve a brother and a sister aged 48 and 60 years, respectively. Both were radiologically diagnosed with pulmonary microlithiasis (Figures 3 and 4), and pulmonary function tests were normal. However, they declined further procedures, including diagnostic bronchoscopy with bronchoalveolar lavage, with or without biopsy, as they were asymptomatic and unwilling to undergo invasive workup and regular follow-up.

## Conclusion

PAM typically progresses slowly and is often diagnosed incidentally through chest X-rays. Confirmation of the diagnosis can be made by identifying characteristic features on a CT scan of the chest. Performing a bronchoscopy and biopsy can further solidify the diagnosis.

## Conflict of Interest

No conflict of interest.
